# Progress in Surgery

**Published:** 1899-11-11

**Authors:** 


### PROGRESS IN SURGERY.

### INJURIES OF THE SPINE.

The principles of treatment of injuries of the spinal cord forms the subject of a paper by Bolton, of New York.[1] Extra-medullary hæmorrhage occurs oftenest between the dura and the wall of the spinal canal, far less often within the dura; but it is yet to be proved whether blood between the dura and the bone ever occurs in sufficient quantity to cause compression.

When symptoms of compression exist there is almost invariably some demonstrable lesion of the cord present.

Complete lesions of the cord are usually not more than an inch in length, but the whole structure of the cord is destroyed; and even if the patient survive no regeneration of the nerve elements ever takes place, the only union being by cicatricial tissue.

Incomplete lesions usually result from stab or bullet wounds, or distortions of the spine. Foreign bodies are often driven into the cord; or hæmorrhages take place chiefly into its grey substance, and particularly at the cervico-dorsal and dorso-lumbar junctions. The author's conclusions with regard to treatment are—(1) That extra-dural hæmorrhage does not give rise to cord lesions or symptoms, and therefore requires no treatment. (2) Total lesions of the cord are irremediable, because the cells and fibres of the entire thickness of the cord are destroyed, are never regenerated, and are replaced by cicatricial tissue. The lesion thus is permanent and requires no treatment. (3) In hæmato-myelia the clot is absorbed, its site persists as a cavity, or is filled by newly-formed tissue; irregularities of circulation in the surrounding portions of the cord adjust themselves. There may be a great amelioration of the symptoms. There is therefore no therapeutic indication and no remedial treatment is possible. (4) In partial contusion of the cord the lesion results in permanent destruction of cells and fibres; disturbances of circulation adjust themselves. Repair is accomplished by cicatricial tissue. No treatment is available. (5) In open injuries of the cord there are destruction of cells and fibres and disturbances of circulation. In addition, infection may occur if a foreign body be introduced and left in or lodged against the cord, and by its continued presence produce great disturbance of circulation and consequent extensive degeneration and necrosis of cells and fibres. Repair also occurs by cicatricial tissue. But here active operative interference is indicated to remove foreign bodies, to facilitate disinfection, to prevent more extensive necrosis, and to facilitate drainage.

**Laminectomy for Dislocation of the Spine.**—The value of this procedure is still much discussed. Johnston[2] reports a very encouraging case. A man, 32, fell from a tree, landing on his buttocks, and became paralysed below the waist. Three months later he had only regained slight power of movement, but was still in a very helpless condition. The last dorsal and first lumbar spines were exposed, and the spines and arches of these vertebræ removed. It was found that the arch of the last dorsal was pressing forward on the dura, which was also compressed by a mass of fibrous cicatricial tissue. On puncturing the dura a quantity of clear cerebro-spinal fluid escaped under some tension. After the operation the patient complained of a good deal of pain in his legs. Within a week he began to micturate voluntarily, and eventually regained complete control over his bladder and bowels. He was able to walk and resume his occupation. The anæsthesia completely disappeared, and his health was restored.

**Injuries of the Cervical Vertebræ.**—An interesting discussion followed the presentation of a case of fracture of the fifth cervical vertebra to the Philadelphia Academy of Surgery by Dr. T. G. Morton.[3] The patient was a man of 35, who dived into shallow water and struck his head on the bottom. He was not rendered unconscious, but there was complete loss of motion and sensation to the trunk and limbs. He lost control of the bladder and bowels. About a month later an operation was performed, and the laminæ of the fifth cervical were found fractured and removed. The membranes were not opened. Although slight improvement followed in certain of the symptoms the patient gradually sank and died exhausted in about a month. At the post-mortem examination the cord was found softened and degenerated at the level of the fifth and sixth cervical vertebræ.

Dr. Holmes reported two similar cases, both fatal without operation.

Dr. Burr referred to a case of fracture of the cervical spine which occurred 20 years ago, and the patient had been able to attend to his work for many years. He died of Bright's disease, and it was found on post-mortem examination that one of the cervical vertebræ was locked over the one next it, and that the cord was markedly flattened, being only about one-half as thick, and as wide as normally, yet it had fulfilled all its functions.

Dr. Deaver, although he has not had much success in operations for spinal injuries, considers the operation justifiable, in view of the almost hopeless prognosis in any case. He thinks the operation should be done at once, and not after prolonged intervals, and in this opinion Dr. Harte agrees with him. His experience of operation also leads him to prefer a conservative line of treatment. Dr. Wharton has so often seen good results follow without operation that he prefers to leave matters to nature, and this is also the position taken up by Dr. T. R. Neilson. Dr. Mears, on the other hand, thinks it not only justifiable, but incumbent on the surgeon to give the patient the benefit of the operation, and he believes that only by repeatedly operating shall we improve our knowledge for diagnostic purposes and obtain better results by treatment.

A new syndrome (group of symptoms) consecutive to injuries of the spinal cord is described by Urriola,[4] *apropos* of a case of stab between the seventh and eighth dorsal vertebræ, the outstanding features of which are (1) a flaccid paraplegia immediately following the wound; (2) complete anæsthesia of the right leg below the level of the hip joint, and hyperæsthesia of the left leg; (3) distinct allochira, which lasted for a week; (4) total loss of deep reflexes during first weeks, returning later, and finally becoming exaggerated; foot clonus, spasticity and contraction varying in the two legs; (5) persistence of a narrow zone of anæsthesia of right leg; and (6) absence of any trophic or vaso-motor symptoms.

**Fractures and Dislocations.**—Dr. W. B. Ferguson records an interesting case[5] in which he succeeded in reducing a partial dislocation of the cervical spine by manipulation, with immediate relief to the urgent symptoms of the patient. Mr. Noble Smith contributes a lengthy paper[6] on fracture-dislocation of the spine, the main object of which is to advocate the use of a particular appliance he has devised for fixation of the spine, on the same lines as that he has for some time used for caries.

[1] Annals of Surgery, Aug., 1899. [2] Ibid. [3] Ibid. [4] Arch. de Neurol., May, 1899; Brit. Med. Jour. Epitome, July 1, 1899. [5] Lancet, July 8, 1899. [6] Lancet, Aug. 19, 1899.

